# Hololens and Vive Pro: Virtual Reality Headsets

**DOI:** 10.5195/jmla.2019.602

**Published:** 2019-01-01

**Authors:** Dorothy Carol Ogdon

**Affiliations:** Assistant Professor, Emerging Technologies Librarian, UAB Libraries, University of Alabama at Birmingham, Birmingham, AL, dogdon@uab.edu

## INTRODUCTION

Virtual reality is “a three-dimensional computer-generated environment, either real-world or imagined, that simulates an intractable sensory experience through the use of specific equipment” [1]. In the past decade, a number of commercial tools for accessing immersive and mixed virtual reality environments have been released. These tools are now entering the higher education ecosystem and are likely to become a common tool for research, instruction, and even health care delivery in the next ten to twenty years [1]. The VIVE Pro from HTC and the HoloLens from Microsoft are two virtual reality headsets that are quickly gaining recognition, although neither has yet to be identified as having a clear advantage over the other in terms of usability in or applicability to educational or clinical settings [2, 3].

## DEVICE DESCRIPTIONS

### VIVE Pro

The VIVE Pro from HTC was released April 5, 2018 [4]. The VIVE Pro is an immersive virtual reality headset that uses two infrared sensors, called base stations, and two handheld controllers to track the motion of users wearing the headset [1, 2, 5, 6]. The headset must be used in range of the base stations to function properly and currently requires a wired connection to a virtual reality–ready computer, though HTC recently began accepting preorders for a wireless adapter for both the VIVE Pro and VIVE virtual reality headsets [7]. The area within range of the base stations is usually referred to as the “play area” and is often marked on the floor in the physical spaces where the headsets are being used [2, 8]. The play area should be kept clear of obstacles, including furniture, while the headset is in use [2, 8]. The VIVE Pro has a larger play area than its predecessor, the HTC VIVE, and includes a front-facing camera, detachable speakers, adjustments for lens and interpupillary distance, and a redesigned headband [9, 10]. The face cushion on the headset can be easily replaced [11].

### Microsoft Hololens

The Microsoft HoloLens is an augmented reality headset that the manufacturer describes as “the leader in mixed reality technology” [1, 3]. The terms “augmented reality” and “mixed reality” refer to an approach to virtual reality that is not totally immersive [1]. Rather than totally immersing a user in a completely virtual visual environment, mixed and augmented reality devices and applications layer interactive images over the physical environment.

Unlike the VIVE Pro, HoloLens uses gesture and gaze (looking at a specific area of the virtual environment) recognition technology and does not require the use of handheld controllers, though a clicker that can be used in place of tap gestures is included with the headset. Users interact with the virtual interface and holograms through a combination of gazing and tapping, grasping, or using the bloom hand gesture to open and close windows and menus [12]. The Cortana virtual assistant and voice command functions are also built in to HoloLens [13]. The headset is designed to operate as a self-contained unit and can be operated through a wireless connection only.

Operation of the HoloLens does not require additional external sensors or a constant connection to a computer, although the device can be managed by connecting it to a desktop through an included USB cable. Users can also pair HoloLens with Bluetooth devices, including wireless keyboards, to facilitate typing and other tasks. HoloLens automatically calibrates pupillary distance and features an adjustable headband. The headset includes built-in speakers and can support two to three hours of battery-powered use [13].

## SPECIFICATIONS AND REQUIRED EQUIPMENT

Both the HoloLens and the VIVE Pro provide a high-resolution display. The HoloLens resolution is 2.4 million total light points, while the VIVE Pro provides a combined 2880 x 1600 pixels [13]. At 579 grams, the HoloLens slightly outweighs the VIVE Pro, which VR Bound reports as weighing 555 grams [13, 14]. Weight and ergonomics are important considerations for virtual reality headsets because they influence how long users can comfortably wear a device.

The VIVE Pro uses Steam VR software and must be continuously connected to a virtual reality–ready computer that meets the minimum specifications for both the headset and any desired software [15, 16]. Use of the VIVE Pro also requires purchasing and operating 2 base stations and 2 handheld controllers [2]. As of September 2018, minimum specifications for a workstation to be used with the VIVE Pro headset are an Intel Core i5-4590 processor and an NVIDIA GeForce GTC 970 graphics card or their equivalents. At least 4 gigabytes (GB) RAM is also required, and Windows 10 is the recommended operating system for best results, though Windows 7 and later systems are supported [2]. HoloLens requires at least 8GB RAM and Windows 10 [17].

## STRENGTHS AND WEAKNESSES

The HoloLens has several advantages over the VIVE Pro in terms of device management, even though the VIVE Pro provides a richer virtual reality experience. The HoloLens does not require a dedicated play area, is self-contained, and does not require handheld controllers. The HoloLens commercial suite provides access to a kiosk mode and device management tools that are integrated into Microsoft Enterprise systems [18, 19]. The HoloLens interface will be familiar to users who use the Windows operating system on other computing platforms, making first-time use of the device easier for users. Though it is self-contained and does not require additional accessories, the commercial license for HoloLens has a much higher price point than the VIVE Pro, even when considering the additional equipment needed to create a play space.

The need for a dedicated play space for the VIVE Pro is a disadvantage for those working with limited physical library space. Though the computer workstation required to operate the VIVE Pro may be a disadvantage in terms of space allocation and space use logistics, the dedicated workstation will facilitate storing large files more easily than the HoloLens, which has 64GB Flash and 2GB RAM onboard memory [13]. The required dedicated virtual reality ready workstation also allows libraries to connect multiple monitors or televisions to a single source, which enables users who are not wearing the headset to view activities taking place in the virtual environment.

## SOFTWARE AND MANAGEMENT

Following device selection and setup, the greatest challenge for libraries interested in providing access to virtual reality tools will be software and device management.

During device setup, the VIVE Pro user instructions direct users to use Steam VR, which is designed primarily for individual users, rather than large organizations or educational institutions. Steam VR provides commercial site licensing through the Steam PC Café program [20]. Libraries should examine the terms of this license carefully when considering this option for virtual reality workstation management. As described on the program website, the Steam PC Café program allows users to access institutional content as well as their personal Steam VR accounts, which is likely to lead to devices being used for purposes other than education and training. Other vendors, such as SpringboardVR, offer management services for virtual reality stations, and interested librarians should expect management and licensing options in this area to change rapidly in the next several years [21].

Use of the HoloLens in professional and educational settings requires the HoloLens commercial suite, which significantly increases the price of the device. There are several options for device management that are supported by the commercial suite, including a kiosk mode and multiple device management that can be implemented using Microsoft enterprise tools [21]. Microsoft provides a rental option for the HoloLens that will be useful for libraries wishing to explore use of these devices without making a long-term commitment to an unfamiliar platform and device.

The promise of virtual reality for simulation, training, and education is vast. Common health sciences–related software content typically covers topics such as human anatomy, overviews of surgical procedures, and occasional tours of programs that are intended to be used for clinical interventions, such as Therapy Lens for the HoloLens [22, 23]. There are also viewer programs available for digital imaging and communications in medicine (DICOM) files [23]. Many of these programs are free or low-cost, making ongoing costs for supporting virtual reality tools easily feasible after the initial purchase of equipment. Interestingly, even though software that addresses similar topics is available on both platforms, there does not yet seem to be cross-platform duplication of software packages. This might be a limiting factor for libraries that wish to offer the same software on different virtual reality platforms. Tables 1 and 2 provide an overview of health sciences–related software that is available for the VIVE Pro and HoloLens.

**Table 1:** Anatomy and health sciences–related software available for the HTC VIVE Pro [15]

Name	Content	Cost
3D Organon VR Anatomy	Human anatomy	Paid
The Body VR: Anatomy Viewer	Human anatomy	Free
MEDICALHOLODECK	View DICOM imaging	Free, paid version available
The Physiology of the Eye	Human anatomy, eye anatomy	Paid
Sharecare VR	Human anatomy, eye anatomy	Free

**Table 2:** Anatomy and health sciences–related applications for the Microsoft HoloLens [24]

Name	Content	Cost
The Cystic Fibrosis-CRISPR Experience	CRISPR/Cas9 genome editing	Free
Dicom Director	View DICOM imaging	Free demo
DynamicAnatomy	Human anatomy, ankle joint	Free
Holo Eye Anatomy	Human anatomy, eye anatomy	Free
HoloAnatomy	Human anatomy academic course content	Free tour
Human eye and cataract	Human anatomy, eye anatomy	Free
Insight Heart	Human anatomy, heart	Free
Learning Heart	Human anatomy, heart	Free
Therapy Lens	Demonstration of mixed reality to support activities of daily living	Free demo

## SUMMARY

Virtual reality devices are heavily used as soon as they are made available in educational environments, even though the management ecosystems, research, and instructional uses for these devices are still in the early stages of development [25]. The utility of these devices to support student learning, training, simulation, and eventually health care delivery should not be underestimated. The intersectional nature of these devices makes them ideally suited for use in the library. By nature of their relationship to issues of access to both technology and information, librarians already possess the knowledge and skills needed to support the introduction and use of these tools by patrons with a widely varying degree of technical skills and knowledge. It will be to the advantage of academic health sciences and other medical libraries to proactively plan to provide access to these devices for educational and research purposes.
